# Real-world clinical burden and economic assessment associated with hyperkalaemia in a large integrated healthcare system: a retrospective analysis

**DOI:** 10.1186/s12875-022-01667-1

**Published:** 2022-04-01

**Authors:** Joseph B. Muhlestein, Jennifer Kammerer, Tami L. Bair, Kirk U. Knowlton, Viet T. Le, Jeffrey L. Anderson, Donald L. Lappé, Heidi T. May

**Affiliations:** 1 Intermountain Medical Center Heart Institute, Salt Lake City, Utah USA; 2 University of Utah, Salt Lake City, Utah USA; 3 Vifor Pharma, Inc, Redwood City, CA USA; 4 Rocky Mountain University of Health Professions, Provo, Utah USA

**Keywords:** Hyperkalaemia, Risk assessment, Heart failure, Health service research, Health resources

## Abstract

**Background:**

Hyperkalaemia (HK) is a serious and potentially life-threatening condition. Both acute and chronic conditions may alter potassium homeostasis. Our aim is to describe HK incidence, clinical outcomes, and associated resource use within a large, integrated healthcare system.

**Methods:**

Adult patients seen at Intermountain Healthcare facilities with a serum potassium (sK) result between January 1, 2003 and December 31, 2018 were retrospectively studied. Descriptive assessment of a population with detected HK, defined by any sK > 5.0 mmol/L and HK frequency and severity to associated resource use and characteristics of HK predictors were made. Multivariable Cox hazard regression was used to evaluate HK to outcomes.

**Results:**

Of 1,208,815 patients included, 13% had HK. Compared to no-HK, HK patients were older (60 ± 18 vs 43 ± 18 years, *P* < 0.001), male (51% vs 41%, *P* < 0.001), and had greater disease burden (Charlson Comorbidity Index 3.5 ± 2.8 vs 1.7 ± 1.4, *P* < 0.001). At 3 years, more HK patients experienced major adverse cardiovascular events (MACEs) (19 vs 3%, *P* < 0.001), persisting post-adjustment (multivariable hazard ratio = 1.60, *P* < 0.001). They incurred higher costs for emergency department services ($552 ± 7,574 vs $207 ± 1,930, *P* < 0.001) and inpatient stays ($10,956 ± 93,026 vs $1,477 ± 21,423, *P* < 0.001). HyperK Risk Scores for the derivation and validation cohorts were: 44% low-risk, 45% moderate-risk, 11% high-risk. Strongest HK predictors were renal failure, dialysis, aldosterone blockers, diabetes, and smoking.

**Conclusion:**

Within this large system, HK was associated with a large clinical burden, affecting over 1 in 10 patients; HK was also associated with increased 3-year MACE risk and higher medical costs. Although risk worsened with more severe or persistently recurring HK, even mild or intermittent HK episodes were associated with significantly greater adverse clinical outcomes and medical costs. The HyperK Score predicted patients who may benefit from closer management to reduce HK risk and associated costs. It should be remembered that our assumptions are valid only for detected HK and not HK per se.

## Background

Hyperkalaemia (HK), typically defined as a serum potassium (sK) concentration > 5 or > 5.5 mmol/L, is a serious medical condition that can lead to life-threatening cardiac arrhythmias and sudden cardiac death [1–3]. It can result from various acute and chronic conditions that affect potassium (K^+^) homeostasis, but commonly occurs in patients with chronic kidney disease (CKD) with comorbidities such as heart failure (HF), diabetes mellitus (DM), and hypertension (HTN) [1–3]. HK is a condition often seen in the emergency department (ED) and may present with noncardiac symptoms (eg, altered mental status, confusion, muscle cramps and weakness, fatigue, paresthesias) [4]. Thus, HK may go unrecognized with few or no obvious symptoms prior to cardiac arrhythmias and/or sudden cardiac arrest [5, 6].

A recent retrospective study evaluating the 5-year prevalence of HK in approximately 1.7 million patients found that 47.6% of patients with CKD (stages 3 and 4) and HF had at least 1 HK event compared to 8.5% of patients without comorbid CKD, end-stage renal disease, HF, or DM [7]. Moreover, optimal treatment regimens for these underlying diseases often include renin–angiotensin–aldosterone system inhibitors (RAASi’s), which further increase HK risk [8]. Furthermore, given the chronic and progressive nature of CKD, patients with or without comorbidities are at long-term risk for HK [3, 7]. HK management options have historically lacked proven efficacy for chronic use (eg, dietary K^+^ intake reduction, loop diuretics, dose reduction, or discontinuation of RAASi’s).

HK incidence and subsequent clinical and economic outcomes within a large healthcare system have not previously been fully described. This study describes the incidence of HK within the Intermountain Healthcare population, assesses associated clinical and economic impacts, and employs a predictive risk tool to identify vulnerable at-risk subsets who may benefit from better HK management.

## Methods

A retrospective, observational database analysis of electronic medical records from Intermountain Healthcare was performed to describe the clinical and economic burden of HK. Intermountain Healthcare is a large, nonprofit, electronically integrated healthcare network consisting of hospitals, clinics, and a system of health insurance plans. Intermountain Healthcare provides services to approximately 65% of the population of Utah, and parts of Idaho and Nevada. This study was approved by the Intermountain Healthcare institutional review board.

In an effort to only include patients from the Intermountain facility that would have complete data and long-term follow-up, patients had to have ≥ 2 nonurgent care or ED encounters at least 2 years apart at an Intermountain facility. Additionally, patients had to be ≥ 18 years of age and have at least one nonspurious sK between January 1, 2003 and December 31, 2018.

Patients were stratified into one of two groups: no-HK (all sK ≤ 5.0 mmol/L) or HK (at least one sK > 5.0 mmol/L). Index date was defined as the first sK measurement received after January 1, 2003 that was ≤ 5.0 mmol/L if in the no-HK group, and first sK measurement received after January 1, 2003 that was > 5.0 mmol/L for the HK group. They were further classified based on HK frequency or severity. Frequency of HK was characterized as transient (1 occurrence of sK > 5.0 mmol/L), intermittent (> 1 occurrence of sK > 5.0 mmol/L, but < 50% of reported results), or persistent (sK > 5.0 mmol/L for > 50% of reported results). Severity categories were based on index sK: mild HK (sK > 5.0–5.5 mmol/L), moderate HK (sK > 5.5–6.4 mmol/L), or severe HK (sK > 6.4 mmol/L). Baseline characteristics assessed included age, sex, traditional cardiovascular (CV) risk factors, prior diagnoses, and medications. Discrete variables were presented as frequencies and continuous variables as means and standard deviations. However, if characteristics were found to be nonnormally distributed, the median and interquartile ranges were reported. The student’s t-test, analysis of variance, and chi-square statistic were used to characterize the populations, and where appropriate, nonparametric tests were used.

Follow-ups at 1 and 3 years were assessed from electronic medical records. Univariable and multivariable logistic and Cox Hazard regression analyses were used for outcomes evaluation. Multivariable models used baseline and clinical characteristics, test results, and medications to adjust for group differences. Final multivariable models retained significant and confounding variables with odds ratios (ORs) and hazard ratios (HRs) reported. A *P*-value of ≤ 0.05 was deemed significant. To determine data accuracy, means and frequencies were compared to prior like-studies for similarity and trends.

Costs, not charges, were determined for each patient encounter using a cost database created internally by Intermountain Healthcare. Total costs included all those associated with outpatient, inpatient, and ED visits. It did not include costs incurred outside of those visits, such as prescription drug costs incurred because medications were filled at a pharmacy after leaving the visit. ED costs included any incurred as a result of that ED visit. It would not include the filling of prescription medications after leaving the ED. Annualized ED and inpatient visits in the follow-up period were quantified and stratified by the presence or absence of HK. Average annual total and ED costs were calculated by summing the costs (in dollars) for each year and dividing by the number of years contributed.

A risk score, termed the HyperK Score, was created to help identify patients who are most at risk of developing HK. Therefore, the patients were randomly divided into two groups: a derivation cohort (70% of the population) where the risk score was created and a validation cohort (30% of the population) where the risk score was applied to determine how well it predicted HK. It was derived by multiplying the β-coefficient for each of the significant and confounding variables, rounding to the nearest integer, and summing the values. The risk score was then stratified into risk categories of low (< 90% sensitivity threshold), moderate (≥ 90% sensitivity threshold but < 90% specificity threshold), and high (≥ 90% specificity threshold). Receiver operator characteristic curves were used to determine the area under the curve c-statistic from risk score data. Overall risk scores were applied to compare c-statistics and associated 95% confidence intervals (CIs).

### Role of the funding source

The sponsor had a role in the study design, conduct of the study, data interpretation, and decision to publish. The findings and conclusions in this article are those of the authors, who are responsible for its contents.

## Results

**Fig1:**
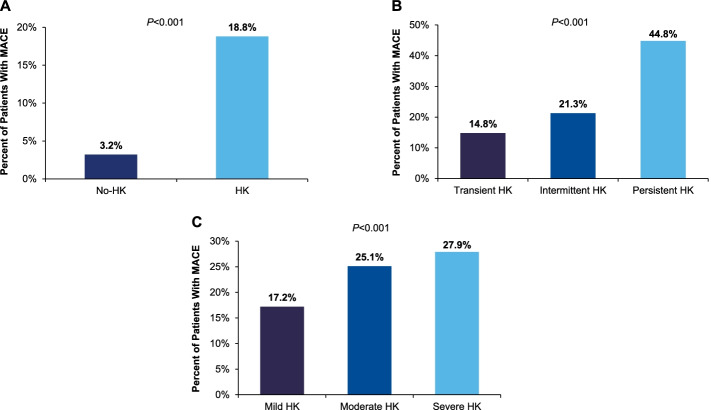
Percent of patients with MACE within 3 years stratified by **A**: HK vs No-HK; **B**: HK frequency; and **C**: HK severity. Figure 1A: The number of patients with 3 years of follow-up was 1,128,582 (no-HK = 983,409, HK = 145,173). Fig. 1B: The number of patients with 3 years of follow-up was 145,173 (transient HK = 73,036, intermittent HK = 67,486, persistent HK = 4,651). Figure 1C: The number of patients with 3 years of follow-up was 145,173 (mild HK = 117,902, moderate HK = 22,879, severe HK = 4,392). *HK* hyperkalaemia; *MACE* major adverse cardiovascular event

A total of 1,208,815 patients met the study criteria, with 161,849 (13%) having detected HK. The average annual incidence was 0.81%. Compared to patients without HK, HK patients were older (mean age 60 ± 18 years vs 43 ± 18 years), more often male (51 vs 41%), had a higher Charlson Comorbidity Index (CCI; 3.5 ± 2.8 vs 1.7 ± 1.4; *P* < 0.001), and were more likely to have renal insufficiency (16.3 vs 0.7%; *P* < 0.001). Atherosclerotic CV disease (CVD), HF, and atrial fibrillation were among the highest prior diagnoses in patients with HK (21, 20, and 14%, respectively) and significantly higher than in the no-HK group (4, 2, and 2%, respectively). Baseline characteristics stratified by the presence of HK are presented in Table 1. At baseline, all assessed medication use was more common in the HK group, K^+^-binder use was low overall (< 1%); sodium polystyrene sulfonate (SPS) was used in 0.4% of patients with HK and 0.04% of patients without HK (*P* < 0.001); and patiromer use was insufficient to evaluate. At 3-year follow-up, major adverse CV events (MACEs) were higher in the HK vs no-HK group (18.8 vs 3.2%; *P* < 0.001), which persisted after adjustment (multivariable HR = 1.60; *P* < 0.001; Table 1, Fig. 1A).

**Table 1:** Overall population – baseline characteristics and post-index outcomes

	**No-HK** ***n* = 1,046,966**	**HK** ***n* = 161,849**		***P*-value**
Mean age (years)	43 ± 18	60 ± 18		< 0.0001
Age categories				< 0.0001
< 65 years	86%	56%		
65–74 years	8%	20%		
≥ 75 years	6%	24%		
Sex (male)	41%	51%		< 0.0001
CCI	1.7 ± 1.4	3.5 ± 2.8		< 0.0001^a^
Mean baseline sK (mmol/L)^b^	4.07 ± 0.39	5.38 ± 0.48		< 0.0001
***Traditional CV Risk Factors***				
Hypertension	20%	63%		< 0.0001
Hyperlipidaemia	17%	55%		< 0.0001
Diabetes	7%	33%		< 0.0001
Smoking	13%	26%		< 0.0001
Renal insufficiency	0.7%	16%		< 0.0001
BMI, kg/m^2^, *n* = 662,888	28.4 ± 7.4	29.7 ± 8.1		< 0.0001
BMI categories (kg/m^2^)				< 0.0001
< 25	36%	29%		
25–29.9	31%	31%		
≥ 30	33%	40%		
Maximum SBP (mm Hg)	139.2 ± 71.6	162.8 ± 73.7		< 0.0001
EF (%)	60.9 ± 10.2	58.0 ± 12.9		< 0.0001
***Prior Diagnoses***				
ASCVD	4%	21%		< 0.0001
CAD	1%	7%		< 0.0001
MI	0.6%	4%		< 0.0001
Stroke	0.3%	2%		< 0.0001
TIA	0.7%	4%		< 0.0001
PVD	0.1%	1%		< 0.0001
Heart failure	2%	20%		< 0.0001
Atrial fibrillation	2%	14%		< 0.0001
***Baseline Medications***				
Statin	7%	39%		< 0.0001
Other anti-diabetic	1%	10%		< 0.0001
Insulin	2%	24%		< 0.0001
Metformin	3%	18%		< 0.0001
Sulfonylurea	1%	11%		< 0.0001
NSAID	39%	62%		< 0.0001
ACEi	8%	39%		< 0.0001
ARB	3%	16%		< 0.0001
Aldosterone inhibitor	0.6%	6%		< 0.0001
Beta-blocker	6%	32%		< 0.0001
Diuretic	9%	42%		< 0.0001
CCB	4%	22%		< 0.0001
Furosemide	3%	24%		< 0.0001
Torsemide	0.1%	1.2%		< 0.0001
SPS	0.04%	0.4%		< 0.0001
Patiromer	0%	0%		–
***3-Year Post-Index Outcomes (n* = *1,128,582)***				
	*n* = 983,409	*n* = 145,173		
MACE^c^	3.2%	18.8%		< 0.0001
Death	2.9%	16.7%		< 0.0001
MI	0.05%	0.2%		< 0.0001
Stroke	0.2%	0.9%		< 0.0001
HFH	0.2%	2.8%		< 0.0001
***Multivariable Hazard Ratio (95% CI) for 3-Year MACE***^**c**^				
No-HK vs HK			1.60 (1.57, 1.63), *P* < 0.0001	
***Post-Index Average Costs/Year (n* = *1,208,815)***				
ED				< 0.0001^a^
Cost ± SD	$207 ± 1,930	$552 ± 7,574		
Median (IQR)	11 (0, 187)	56 (0, 408)		
Inpatient				< 0.0001^a^
Cost ± SD	$1,477 ± 21,423	$10,956 ± 93,026		
Median (IQR)	0 (0, 740)	0 (0, 4585)		
***Age* ≥ *60 years Post-Index Average Costs/Year (n* = *290,972)***				
ED				< 0.0001^a^
Cost ± SD	$222 ± 2,747	$561 ± 7,098		
Median (IQR)	18 (0, 219)	72 (0, 444)		
Inpatient				< 0.0001^a^
Cost ± SD	$3,765 ± 35,137	$13,844 ± 109,257		
Median (IQR)	0 (0, 2307)	1,277 (0, 6678)		

*ACEi* Angiotensin-converting enzyme inhibitor, *ARB* Angiotensin-II receptor blockers, *ASCVD* Atherosclerotic cardiovascular disease, *BMI* Body mass index, *CAD* Coronary artery disease, *CCB* Calcium channel blocker, *CCI* Charlson Comorbidity Index, *CV* Cardiovascular, *ED* Emergency department, *EF* Ejection fraction, *HFH* Heart failure hospitalization, *HK* Hyperkalaemia, *IQR* Interquartile range, *MACE* Major adverse CV event, *MI* Myocardial infarction, *NSAID* Nonsteroidal anti-inflammatory drug, *PVD* Peripheral vascular disease, *SBP* Systolic blood pressure, *SD* Standard deviation, *sK* Serum potassium, *SPS* Sodium polystyrene sulfonate, *TIA* transient ischaemic attack

Models adjusted by baseline characteristics, risk factors, and medications

^a^The non-parametric Mann–Whitney rank sum test was utilized to determine *P*-value

^b^Taken within ± 1 month of index date (baseline sK level)

^c^MACE is the composite of death, MI, stroke, and HFH

Table 2 describes baseline characteristics and outcomes by HK frequency. Persistent HK represented only 3% of the total HK population, whereas a large majority showed either intermittent or transient patterns (45 and 52%, respectively). Relative to other HK patterns, persistent HK was more common in older patients (mean age 68 ± 17 years) and was the dominant pattern (40%) in those age ≥ 75 years. Persistently recurring HK was also associated with the highest CCI (4.3 ± 3.1) as well as greater rates of renal insufficiency (30%) and HF (30%). At baseline and relative to more frequent patterns, all medication use was generally lowest among transient HK, with the exception of nonsteroidal anti-inflammatory drugs (NSAIDs). SPS use was low in each group (0.1% of transient HK, 0.7% of intermittent HK, 1% of persistent HK). Substantially higher than other patterns, 3-year MACE was highest (44.8%) with persistently recurring HK, achieving statistical significance (persistent vs transient HK multivariable HR = 2.31 [95% CI, 2.20, 2.43], *P* < 0.001). A smaller difference was observed between less frequently occurring patterns (intermittent vs transient HK multivariable HR = 1.02 [95% CI, 0.99, 1.05], *P* = not significant; Table 2, Fig. 1B). Each of the four individual MACE components (death, myocardial infarction [MI], stroke, and HF hospitalization) influenced risk.

**Table 2:** HK frequency – baseline characteristics and post-index outcomes

	**Transient** ***n* = 82,307 (52%)**	**Intermittent** ***n* = 72,382 (45%)**	**Persistent** ***n* = 5,150 (3%)**	***P*-value**
Mean age (years)	57 ± 19	63 ± 16	68 ± 17	< 0.0001
Age categories				< 0.0001
< 65 years	64%	48%	40%	
65–74 years	16%	24%	20%	
≥ 75 years	20%	28%	40%	
Sex (male)	49%	53%	56%	< 0.0001
CCI	3.1 ± 2.7	3.8 ± 2.8	4.3 ± 3.1	< 0.0001^a^
Mean baseline sK (mmol/L)^b^	5.3 ± 0.5	5.4 ± 0.5	5.6 ± 0.6	< 0.0001
***Traditional CV Risk Factors***				
Hypertension	52%	74%	73%	< 0.0001
Hyperlipidaemia	49%	63%	55%	< 0.0001
Diabetes	23%	43%	43%	< 0.0001
Smoking	24%	29%	27%	< 0.0001
Renal insufficiency	10%	23%	30%	< 0.0001
BMI categories (kg/m^2^)				< 0.0001
< 25	31%	27%	32%	
25–29.9	32%	30%	28%	
≥ 30	37%	43%	40%	
Maximum SBP (mm Hg)	158 ± 82	168 ± 64	168 ± 49	< 0.0001
EF (%)	58 ± 13	57 ± 14	58 ± 13	< 0.0001
***Prior Diagnoses***				
ASCVD	15%	26%	25%	< 0.0001
CAD	5%	10%	9%	< 0.0001
MI	3%	5%	4%	< 0.0001
Stroke	1%	2%	2%	< 0.0001
TIA	3%	4%	4%	< 0.0001
PVD	0.7%	1%	1%	< 0.0001
Heart failure	13%	27%	30%	< 0.0001
Atrial fibrillation	10%	17%	19%	< 0.0001
***Baseline Medications***				
Statin	33%	46%	41%	< 0.0001
Other anti-diabetic	6%	13%	14%	< 0.0001
Insulin	17%	32%	33%	< 0.0001
Metformin	13%	22%	23%	< 0.0001
Sulfonylurea	7%	16%	18%	< 0.0001
NSAID	61%	65%	59%	< 0.0001
ACEi	30%	47%	48%	< 0.0001
ARB	13%	20%	18%	< 0.0001
Aldosterone inhibitor	25%	39%	37%	< 0.0001
Beta-blocker	4%	9%	8%	< 0.0001
Diuretic	33%	52%	50%	< 0.0001
CCB	17%	27%	28%	< 0.0001
Furosemide	17%	32%	34%	< 0.0001
Torsemide	0.7%	2%	2%	< 0.0001
SPS	0.1%	0.7%	1%	< 0.0001
Patiromer	0%	0%	0%	–
***3-Year Post-Index Outcomes (n* = *145,173)***				
	*n* = 73,036	*n* = 67,486	*n* = 4,651	
MACE^c^	14.8%	21.3%	44.8%	< 0.0001
Death	14.1%	17.7%	44.2%	< 0.0001
MI	0.1%	0.3%	0.04%	< 0.0001
Stroke	0.6%	1.2%	0.5%	< 0.0001
HFH	0.9%	5%	1.8%	< 0.0001
***Multivariable Hazard Ratio (95% CI) for 3-Year MACE***^***c***^				
Persistent vs transient HK		2.31 (2.20, 2.43), *P* < 0.0001		
Intermittent vs transient HK		1.02 (0.99, 1.05), *P* = 0.08		
***Post-Index Average Costs/Year***				
ED				
Cost ± SD	$422 ± 4,221	$620 ± 2,305	$1,737 ± 37,846	< 0.0001^a^
Median (IQR)	0 (0, 274)	165 (0, 580)	0 (0, 70)	
1-year visit (primary diagnosis)	0.01%	0.3%	0.7%	< 0.0001^a^
Inpatient				
Cost ± SD	$5,047 ± 51,691	$16,478 ± 108,826	$30,011 ± 245,519	< 0.0001^a^
Median (IQR)	0 (0, 1347)	2681 (0, 9661)	0 (0, 1488)	
1-year visit (primary diagnosis)	0.004%	0.3%	0.9%	< 0.0001^a^

*ACEi* Angiotensin-converting enzyme inhibitor, *ARB* Angiotensin-II receptor blockers, *ASCVD* Atherosclerotic cardiovascular disease, *BMI* Body mass index, *CAD* Coronary artery disease, *CCB* Calcium channel blocker, *CCI* Charlson Comorbidity Index, *CV* Cardiovascular, *ED* Emergency department, *EF* Ejection fraction, *HFH* Heart failure hospitalization, *HK* Hyperkalaemia, *IQR* Interquartile range, *MACE* Major adverse CV event, *MI* Myocardial infarction, *NSAID* Nonsteroidal anti-inflammatory drug, *PVD* Peripheral vascular disease, *SBP* Systolic blood pressure, *SD* Standard deviation, *sK* Serum potassium, *SPS* Sodium polystyrene sulfonate, *TIA* Transient ischaemic attack

Models adjusted by baseline characteristics, risk factors, and medications

^a^The non-parametric Mann–Whitney rank sum test was utilized to determine *P*-value

^b^Taken within ± 1 month of index date (baseline sK level)

^c^MACE is the composite of death, MI, stroke, and HFH

Table 3 identifies baseline characteristics and post-index outcomes by HK severity. Of total HK patients, only 3% had severe HK; most had mild (81%) or moderate (16%) HK. Severity patterns were similar across age groups. Moderate and severe HK were associated with higher baseline disease burden based on CCI. At baseline, SPS use was low regardless of HK severity (0.8% each of moderate and severe HK, 0.3% of mild HK). MACE risk at 3 years positively correlated with HK severity; multivariable HR was 1.31 (95% CI, 1.23, 1.40; *P* < 0.001) for severe vs mild groups and 1.29 (95% CI, 1.25, 1.33; *P* < 0.001) for moderate vs mild groups (Table 3, Fig. 1C). Death and HF hospitalization were the primary drivers of MACE risk.

**Table 3:** HK severity – baseline characteristics and post-index outcomes

	**Mild HK *n* = 131,509 (81%)**		**Moderate HK *n* = 25,380 (16%)**	**Severe HK** ***n *= 4,960 (3%)**	***P*-value**
Mean age (years)	60 ± 18		60 ± 19	58 ± 19	< 0.0001
Age categories					< 0.0001
< 65 years	56%		55%	59%	
65–74 years	20%		19%	18%	
≥ 75 years	24%		26%	23%	
Sex (male)	51%		50%	48%	< 0.0001
CCI	3.2 ± 2.7		3.9 ± 2.9	4.1 ± 3.0	< 0.0001^a^
Mean baseline sK (mmol/L)^b^	5.2 ± 0.1		5.8 ± 0.2	7.3 ± 1.1	< 0.0001
***Traditional CV Risk Factors***					
Hypertension	62%		65%	65%	< 0.0001
Hyperlipidaemia	56%		51%	47%	< 0.0001
Diabetes	32%		36%	37%	< 0.0001
Smoking	25%		29%	33%	< 0.0001
Renal insufficiency	14%		24%	38%	< 0.0001
BMI categories (kg/m^2^)					< 0.0001
< 25	29%		31%	30%	
25–29.9	31%		29%	30%	
≥ 30	40%		40%	40%	
Maximum SBP (mm Hg)	162 ± 77		166 ± 59	166 ± 52	< 0.0001
EF (%)	38 ± 13		57 ± 14	58 ± 13	< 0.0001
***Prior Diagnosis***					
ASCVD	20%		22%	19%	< 0.0001
CAD	7%		8%	7%	< 0.0001
MI	3%		4%	3%	< 0.0001
Stroke	1%		2%	2%	< 0.0001
TIA	4%		4%	3%	0.001
PVD	1%		1%	0.9%	< 0.0001
Heart failure	18%		24%	26%	< 0.0001
Atrial fibrillation	13%		16%	17%	< 0.0001
***Baseline Medications***					
Statin	40%		37%	33%	< 0.0001
Other anti-diabetic	10%		10%	9%	0.55
Insulin	23%		29%	31%	< 0.0001
Metformin	18%		17%	16%	0.01
Sulfonylurea	11%		12%	10%	< 0.0001
NSAID	63%		61%	60%	< 0.0001
ACEi	38%		39%	36%	< 0.0001
ARB	16%		16%	15%	0.10
Aldosterone inhibitor	6%		8%	9%	< 0.0001
Beta-blocker	31%		34%	32%	< 0.0001
Diuretic	41%		46%	45%	< 0.0001
CCB	21%		24%	23%	< 0.0001
Furosemide	23%		29%	30%	< 0.0001
Torsemide	1%		2%	1%	< 0.0001
SPS	0.3%		0.8%	0.8%	< 0.0001
Patiromer	0%		0%	0%	–
***3-Year Post-Index Outcomes (n* = *145,173)***					
	*n* = 117,902		*n* = 22,879	*n* = 4392	
MACE^c^	17.2%		25.1%	27.9%	< 0.0001
Death	15.2%		22.7%	25.9%	< 0.0001
MI	0.2%		0.2%	0.2%	0.79
Stroke	0.9%		1%	0.7%	0.06
HFH	2.6%		3.6%	3.3%	< 0.0001
***Multivariable Hazard Ratio (95% CI) for 3-Year MACE***^**c**^					
Moderate vs mild HK			1.29 (1.25, 1.33), *P* < 0.0001		
Severe vs mild HK			1.31 (1.23, 1.40), *P* < 0.0001		
***Post-Index Annualized Acute Care Trends***					
ED					
Mean cost ± SD		$519 ± 7,819	$701 ± 6,936	$668 ± 2,100	< 0.0001^a^
Median (IQR)		54 (0, 391)	67 (0, 477)	68 (0, 554)	
1-year visit (primary diagnosis)		0.1%	0.4%	0.7%	< 0.0001^a^
Inpatient					
Mean cost ± SD		$9,626 ± 73,418	$16,328 ± 155,262	$18,743 ± 125,932	< 0.0001^a^
Median (IQR)		0 (0, 4235)	0 (0, 6222)	788 (0, 7332)	
1-year visit (primary diagnosis)		0.1%	0.4%	1.1%	< 0.0001^a^

*ACEi* Angiotensin-converting enzyme inhibitor, *ARB* Angiotensin-II receptor blockers, *ASCVD* Atherosclerotic cardiovascular disease, *BMI* Body mass index, *CAD* Coronary artery disease, *CCB* Calcium channel Blocker, *CCI* Charlson Comorbidity Index, *CV* Cardiovascular, *ED* Emergency department, *EF* Ejection fraction, *HFH* Heart failure hospitalization, *HK* Hyperkalaemia, *IQR* Interquartile range, *MACE* Major adverse CV event, *MI* Myocardial infarction, *NSAID* Nonsteroidal anti-inflammatory drug, *PVD* Peripheral vascular disease, *SBP* Systolic blood pressure, *SD* Standard deviation, *sK* Serum potassium, *SPS* Sodium polystyrene sulfonate, *TIA* Transient ischaemic attack

Models adjusted by baseline characteristics, risk factors, and medications

^a^The non-parametric Mann–Whitney rank sum test was utilized to determine *P*-value

^b^Taken within ± 1 month of index date (baseline sK level)

^c^MACE is the composite of death, MI, stroke, and HFH

ED and inpatient costs in the HK and no-HK groups were compared (Table 1), with substantial divergence of medians and median ranges in older patients. In the overall HK population, average annual costs were $552 ± 7,574 for ED and $10,956 ± 93,026 for inpatient visits; in the no-HK group, costs were $207 ± 1,930 for ED and $1,477 ± 21,423 for inpatient visits. Notably, in patients age ≥ 60 years, average annual ED and inpatient costs were significantly higher in HK patients ($561 ± 7,098 for ED and $13,844 ± 109,257 for inpatient) relative to those without HK ($222 ± 2,747 for ED and $3,765 ± 35,137 for inpatient) (Table 1).

Higher HK frequency correlated with greater ED and inpatient costs (Table 2), with greatest impact on inpatient costs. For transient, intermittent, and persistent HK patterns, ED costs were $422 ± 4,221, $620 ± 2,305, and $1,737 ± 37,846, respectively; and inpatient costs were $5,047 ± 51,691, $16,478 ± 108,826, and $30,011 ± 245,519, respectively. For patients with a primary diagnosis of HK, visits within 1 year increased in parallel with greater HK frequency.

Increasing severity showed similar trend patterns but with lower magnitudes of cost increases (Table 3). The severe-HK group incurred the highest mean annual inpatient costs ($18,743 ± 125,932; *P* < 0.001) and 1-year hospitalization rate (1.1%; *P* < 0.001). The moderate-HK group averaged the highest annual ED costs ($701 ± 6,936; *P* < 0.001), albeit a modest numeric difference from other groups.

The general trends suggest that inpatient costs trended upward with both severity and frequency of HK, while ED costs trended upward with frequency but not severity. Persistently recurring HK diverged from other frequencies and any severity with regard to impact on both ED and inpatient costs.

A HyperK Risk Score was calculated for a total of 1,077,306 patients using clinical risk predictors from standard factors from the electronic health record. Baseline characteristics among the derivation and validation cohorts are shown in Table 4. Of patients who were given HyperK Risk Scores, 754,109 (70.0%) were in the derivation cohort. Within the derivation cohort, 732,936 (97.2%) did not have HK (defined as sK < 5.0 mmol/L) and 21,173 (2.8%) had HK (defined as sK > 5.5 mmol/L). Among the derivation cohort, 334,504 (44.4%) were considered low-risk, 337,335 (44.7%) were moderate, and 82,270 (10.9%) were high-risk. 323,197 (30.0%) were applied in a validation cohort, of which 314,030 (97.2%) did not have HK and 9,167 (2.8%) had HK (sK > 5.5 mmol/L). Patients with an intermediate sK between > 5.0 and 5.5 mmol/L were excluded. In the validation cohort, 143,328 (44.3%) were low-risk, 144,990 (44.9%) were moderate, and 34,879 (10.8%) were high-risk.

**Table 4:** HyperK – baseline characteristics

	**Derivation** *n* = 754,109	**Validation** *n* = 323,197
Mean age (years)	43.1 ± 18.4	43.0 ± 18.4
< 65 years	85.1%	85.2%
65–74 years	8.4%	8.3%
≥ 75 years	6.5%	6.5%
Sex (male)*	41.7%	41.5%
Hypertension	21.5%	21.4%
Hyperlipidaemia	18.0%	17.9%
Diabetes	7.4%	7.3%
Smoking	13.0%	12.9%
ASCVD	4.2%	4.1%
Heart failure	2.6%	2.6%
Atrial fibrillation	2.5%	2.5%
Renal failure	1.5%	1.5%
Dialysis*	0.2%	0.2%
ACEi	5.6%	5.6%
ARB	2.2%	2.2%
Beta-blocker	4.5%	4.5%
Aldosterone	0.6%	0.6%
NSAID	25.3%	25.3%
Furosemide	2.2%	2.3%
Torsemide	0.1%	0.1%

*ACEi* Angiotensin-converting enzyme inhibitor, *ARB* Angiotensin-II receptor blockers, *ASCVD* Atherosclerotic cardiovascular disease, *NSAID* Nonsteroidal anti-inflammatory drug

^*^P < 0.05

**Fig. 2:**
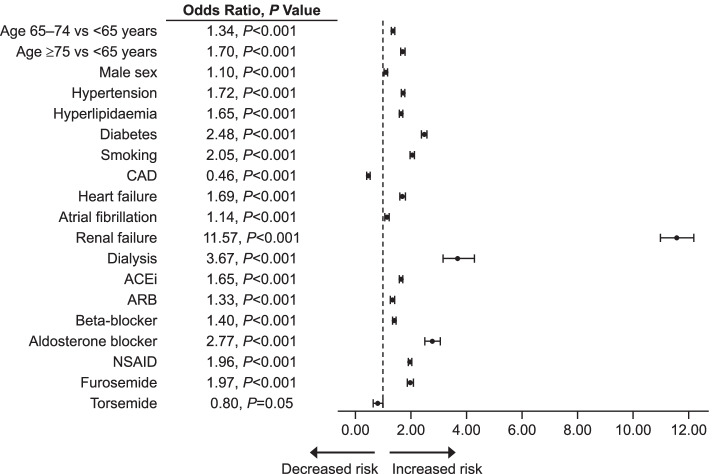
Significant predictors of incident HK (sK > 5.5 mmol/L) compared to No-HK (sK ≤ 5.0 mmol/L). *ACEi* angiotensin-converting enzyme inhibitor; *ARB* angiotensin-II receptor blocker; *CAD* coronary artery disease; *HK* hyperkalaemia; *NSAID* nonsteroidal anti-inflammatory drug; *sK* serum potassium

Significant predictors of HK (Fig. 2) as validated within this population are consistent with other bodies of evidence and include advancing age, male gender, comorbidities (eg, DM, HTN, HF, renal failure), and medications associated with altered K^+^ homeostasis (eg, RAASi’s, diuretics, beta-blockers, NSAIDs). Area under the curve c-statistics and 95% CIs for the derivation and validation cohorts were 0.850 (0.847, 0.853) and 0.853 (0.848, 0.858), respectively. ORs for incident HK in patients in the derivation cohort were evaluated in moderate- vs low-risk (OR = 2.64; *P* < 0.001) and high- vs low-risk (OR = 32.49; *P* < 0.001). In the validation cohort, OR for incident HK was evaluated in moderate- vs low-risk (OR = 2.81; *P* < 0.001) and high- vs low-risk (OR = 34.79; *P* < 0.001).

## Discussion

Prior to this study, HK incidence and subsequent clinical and economic outcomes within a large healthcare system had not been fully described. Within Intermountain Healthcare, HK was associated with a large clinical burden, involving more than 1 in 10 patients.

In the general population, HK is not common [9]. However, this may be because it is a transient condition and so without ongoing monitoring and very limited prospective clinical trials, the true incidence may be underestimated. Additionally, different thresholds have been used to define HK, with HK incidence being reported as 2–3.5% (> 5.5 mEq/L) [10, 11] and 4.9–11% (> 5.0 mEq/L) [12, 13]. Therefore, further research is needed to more accurately estimate HK incidence.

In this analysis, compared to patients without HK, patients with HK were older (60 ± 18 vs 43 ± 18 years), more often male (51% vs 41%), and had a higher CCI (3.5 ± 2.8 vs 1.7 ± 1.4). Our findings are consistent with those in the medical literature, showing that HK is associated with multiple comorbidities. A retrospective analysis showed that the odds of HK were 57% higher in patients with ≥ 2 comorbid conditions (defined by the CCI criteria) compared with those without comorbidities [3]. Another study showed that in patients with HK, approximately 20.1% also had advanced CKD, 52.8% had DM, 53.3% had coronary artery disease, and 12.6% had peripheral vascular disease; furthermore, advanced CKD was a significant predictor of HK in patients with CVD, which was defined as the presence of HTN and HF [14]. In a retrospective claims analysis, Fitch et al. found that the prevalence of several serious conditions (ie, CKD, HTN, and DM) was markedly higher in patients with HK than in the total Medicare and total commercially insured populations. In the total Medicare population and in Medicare patients with HK, the prevalences of CKD were 11.3% and 64.8%, respectively. In the total commercially insured population and in commercially insured patients with HK, the prevalences of CKD were 0.4% and 31.8%, respectively [15].

This analysis showed that HK was associated with higher yearly ED and inpatient costs than those without HK. These results are consistent with previous studies that evaluated resource utilization of HK [16]. In a study of patients with severe HK, the in-hospital mortality rate was 30.7%, which was strongly correlated with the difference between sK levels at admission and the level at its highest point (OR = 1.83; *P* < 0.001). In 2012, Fitch et al. found that the average allowed per-patient per-month cost was 5 times higher in Medicare patients with HK than in the total Medicare population ($5,645 vs $1,035, respectively) and 15 times higher in commercially insured patients with HK than in the total commercially insured sample [15]. It is not unexpected that the costs are higher in the HK group given the differences in comorbid conditions.

This study showed that HK was associated with a two-thirds increase in 3-year MACE, the composite of death, MI, stroke, and HF hospitalization. At 3 years, more HK patients experienced a MACE (19 vs 3%; *P* < 0.001), which persisted after adjustment (multivariable HR = 1.60; *P* < 0.001). Similarly, Luo et al. showed that high sK levels are associated with a higher risk for hospitalization, MACE, and mortality. Compared with sK levels of 4.5–4.9 mmol/L, the adjusted MACE rate was nearly twofold higher for sK levels ≥ 6.0 mmol/L [17]. Jain et al. showed that in patients with CVD who were taking antihypertensive drugs, HK was associated with increased all-cause mortality and an increased hospitalization rate [14]. In a retrospective observational study of 39,705 adults receiving inpatient critical care, HK was associated with higher all-cause mortality 30 days after the initiation of critical care [18]. Fitch et al. found that the mortality rate in a Medicare sample with HK was 5.5 times higher than that in the total Medicare population (23.5% vs 4.3%, respectively) [15]. In a retrospective study of 1,924 patients diagnosed with acute MI, the mortality risk over a 3-year follow-up period was greater in those with HK than normokalaemia during hospitalization (HR = 4.78 for sK ≥ 5.0 mmol/L) [19]. This information emphasizes that the incidence of HK, and its associated morbidity and mortality, is high enough that it justifies special consideration. Particularly among patients—and perhaps particularly older patient populations—who experience frequent recurrent episodes of HK, appropriate chronic preventative therapy may be considered.

The HyperK Score in this analysis was created and internally validated to have substantial ability to discriminate moderate vs severe HK. Use of the HyperK Score may assist in identifying patients needing careful monitoring and clinical management to prevent HK. This analysis showed that although risk worsens with the severity of HK, even mild HK was associated with a significant increase in medical costs and clinical risk.

### Limitations

This is an observational study of usual care and did not guide treatment standardization, which could affect HK occurrence as well as its severity and frequency. It should be remembered that our assumptions are valid only for detected HK and not HK per se.

Confounding was minimized through statistical modelling and appropriate adjustment. There is also the possibility that not all events were captured if patients experienced events and had to receive care outside of an Intermountain facility. Nutritional intake was not assessed and may affect HK. The population was geographically concentrated in or near Utah and may not represent a general nationwide population. A direct association between the cause and effect of each HK episode cannot be determined from presently available data. In this study, overall rates of use of any K^+^ binder were insufficiently low to analyse.

## Conclusion

This analysis described HK incidence and subsequent clinical and economic outcomes within a large healthcare system. HK was shown to be associated with a large clinical and economic burden, involving more than 1 in 10 patients. HK was also associated with a marked increase in yearly ED and inpatient costs, and a two-thirds increase in 3-year MACEs. Risk, sequelae, and resource use worsened with either HK severity or HK frequency, with persistently recurring HK having perhaps the largest impact. Further, per investigator opinion, chronic management of HK is infrequently done, which was validated in this study. These findings justify a more careful look at mitigating the risks of and active management approaches to HK, including the role of recently approved K^+^ binders for chronic HK management.

## Data Availability

All data generated or analysed during this study are included in this published article.

## Abbreviations

ACEi: Angiotensin-converting enzyme inhibitor
ARB: Angiotensin-II receptor blockers
ASCVD: Atherosclerotic cardiovascular disease
BMI: Body mass index
CAD: Coronary artery disease
CCB: Calcium channel blocker
CCI: Charlson Comorbidity Index
CI: Confidence interval
CKD: Chronic kidney disease
CV: Cardiovascular
CVD: Cardiovascular disease
DM: Diabetes mellitus
ED: Emergency department
EF: Ejection fraction
HF: Heart failure
HFH: Heart failure hospitalization
HK: Hyperkalaemia
HR: Hazard ratio
HTN: Hypertension
IQR: Interquartile range
K^+^: Potassium
MACE: Major adverse cardiovascular event
MI: Myocardial infarction
NSAID: Nonsteroidal anti-inflammatory drug
OR: Odds ratio
PVD: Peripheral vascular disease
RAASi: Renin-angiotensin-aldosterone system inhibitor
SBP: Systolic blood pressure
SD: Standard deviation
sK: Serum potassium
SPS: Sodium polystyrene sulfonate
TIA: Transient ischaemic attack
